# Overexpression of Poplar *PtrWRKY89* in Transgenic *Arabidopsis *Leads to a Reduction of Disease Resistance by Regulating Defense-Related Genes in Salicylate- and Jasmonate-Dependent Signaling

**DOI:** 10.1371/journal.pone.0149137

**Published:** 2016-03-28

**Authors:** Yuanzhong Jiang, Li Guo, Rui Liu, Bo Jiao, Xin Zhao, Zhengyi Ling, Keming Luo

**Affiliations:** 1 Key Laboratory of Eco-environments of Three Gorges Reservoir Region, Ministry of Education, Institute of Resources Botany, School of Life Sciences, Southwest University, Chongqing, 400715, China; 2 State Key Laboratory of Forest Genetics and Tree Breeding, Chinese Academy of Forestry, Beijing, 100091, PR China; 3 Key Laboratory of Adaptation and Evolution of Plateau Biota, Northwest Institute of Plateau Biology, Chinese Academy of Sciences, 810008, Xining, China; Key Laboratory of Horticultural Plant Biology (MOE), CHINA

## Abstract

The plant hormones jasmonic acid (JA) and salicylic acid (SA) play key roles in plant defenses against pathogens and several WRKY transcription factors have been shown to have a role in SA/JA crosstalk. In a previous study, overexpression of the poplar WRKY gene *PtrWRKY89* enhanced resistance to pathogens in transgenic poplars. In this study, the promoter of *PtrWRKY89* (*ProPtrWRKY89*) was isolated and used to drive *GUS* reporter gene. High GUS activity was observed in old leaves of transgenic *Arabidopsis* containing *ProPtrWRKY89-GUS* construct and *GUS* expression was extremely induced by SA solution and SA+MeJA mixture but not by MeJA treatment. Subcellular localization and transactivation assays showed that PtrWRKY89 acted as a transcription activator in the nucleus. Constitutive expression of *PtrWRKY89* in *Arabidopsis* resulted in more susceptible to *Pseudomonas syringae* and *Botrytis cinerea* compared to wild-type plants. Quantitative real-time PCR (qRT-PCR) analysis confirmed that marker genes of SA and JA pathways were down-regulated in transgenic *Arabidopsis* after pathogen inoculations. Overall, our results indicated that PtrWRKY89 modulates a cross talk in resistance to *P*. *syringe* and *B*. *cinerea* by negatively regulating both SA and JA pathways in *Arabidopsis*.

## Introduction

Salicylic acid (SA), synthesized either through enzymatic reactions catalyzed by isochorismate synthase (ICS) and isochorimate pyruvate lyase [1, 2] or from cinnamate in an enzymatic reaction catalyzed by phenylalanine ammonia lyase (PAL) [3, 4], plays an important for plant defense *via* activating systemic acquired resistance (SAR). Invasion of biotrophic or hemibiotrophic pathogens causes synthesis and accumulation of SA in plants. Sequentially changing the cellular redox potential results in the NONEXPRESSOR OF PR1 (NPR1) oligomer transformed to monomer, which translocated into the nucleus and collaborated with TGA transcription factors to activated SA-responsive genes, such as pathogenesis-related gene (*PR1*)[5, 6]. On the other hand, jasmonic acid (JA) and its derivates are essential signaling molecules which regulate the plant response to necrotrophic fungi [7]. JA-Ile is the most bioactive form of JA and are synthesized by JASMONATE RESISTANT 1 (JAR1) in *Arabidopsis thaliana* [8]. These ligand molecules JA–Ile directly induces COI1 protein binding to the Jas domain of JAZ proteins whose consequent degradation releases MYC2 and other transcription factors to invoke the expression of JA-mediated defense related genes, such as *PDF1*.*2* [9, 10].

Plant defense mechanisms against various microbial diseases are extremely complex and are mostly regulated by SA and JA signaling network [11]. Increasing evidences showed the SA and JA signaling sectors usually act antagonistically [12]. In mutant *npr1-1* plants, the repression of SA on *PDF1*.*2* and *VSP2* transcription was completely abolished, which are the markers of JA pathway [13, 14]. The SA pathway also inhibits JA signaling through a negative effect on the ORA59 protein which is a transcription activator of JA-responsive genes [15]. Even some pathogens can release certain chemicals to manipulate these two pathways and win the battle. For instance, JA-mimicking phytotoxin coronatine yielded by virulent bacteria *Pseudomonas syringae*, boosts virulence by inhibiting effectual SA-dependent defenses in plant [16, 17]. On the other hand, synergistic interactions between SA and JA signaling have also been described. For example, *Arabidopsis* treated with low concentrations of SA and JA induced a synergistic effect on the SA- and JA-responsive genes *PR1* and *PDF1*.*2* [18]. Further studies showed that the outcome of the SA-JA interaction was also relied on the timing and sequence of initiation of SA and JA signaling besides relative their concentrations [18, 19]. The antagonist and synergetic interactions of SA and JA signaling are orchestrated *via* complicated regulation network [20]. A number of transcription factors participate in this signal transduction processes. In the presence of SA, class II TGA activate SA signaling pathway, but meanwhile interacts with GRX480 to suppresses JA-responsive genes [21]. *Arabidopsis* MYB44 positively regulates expression of SA-mediated genes and negatively regulates JA-responsive genes [22]. Additionally, several WRKY family members are identified to be involved in SA/JA crosstalk [23–25].

WRKY transcription factors, formed one of the largest family in plants, exert their function in transcriptional reprogramming to response to a variety of pathogen invasion [26]. All WRKY proteins contain one or two highly conserved 60 amino acids WRKY domain consist of heptapeptide WRKYGQK signature and a novel zinc finger motif, both of which are important for WRKY proteins binding in high affinity to the *cis*-elements named W box (TTGACT/C) which distribute in the promoters of the target genes [27, 28]. At the aspect of interaction regulation of SA/JA pathways, several W boxes were found in the promoters of the signaling nodes, indicating that WRKY proteins modulate these two signaling pathways directly. For example, the presence of multiple W boxes in the promoter of (NONEXPRESSER OF PR GENE 1) *NPR1*, which was a key node of SA pathway, indicated the involvement of SA-mediated JA repression [13, 14, 29]. Overexpression of some WRKY genes was also demonstrated to affect the ability of defense mediated by SA and JA pathways. For example, AtWRKY4 enhanced resistance to necrotrophic fungi and played a negative role on plant resistance to biotrophic pathogens [30]. AtWRKY33 modulated the antagonistic effect between defense pathways mediating responses to *Pseudomonas syringae* and necrotrophic pathogens, such as *Botrytis cinerea* [31]. Overexpressing *AtWRKY70* activated SA-induced genes and repressed JA-responsive genes [24]. In addition, three structurally-related WRKY proteins AtWRKY18, AtWRKY40, and AtWRKY60 formed both homocomplexes and heterocomplexes to regulated expressions of SA-regulated *PR1* and JA-regulated *PDF1*.*2* and DNA binding activities were impressively shifted relying on which WRKY members were present in the complexes [32]. Most researches have shown the antagonistic regulations between SA- and JA-dependent pathways *via* WRKY transcription factors, but there were limited evidences that WRKY protein may be involved in synergetic interaction of SA- and JA-mediated plant defense. In the *Atwrky70* mutant, both the SA-responsive *PR*s (*PR1* and *PR2*) and the JA-induced *PDF1*.*2* had been elevated compared to wild-type plant [33]. However, the mechanism of WRKY proteins regulating the synergetic effects between SA- and JA-dependent signaling pathways remains unknown.

In previous study, we isolated a WRKY Group III member *PtrWRKY89* from *Populus trichocarpa*, which was rapidly induced by SA, and overexpression of *PtrWRKY89* resulted in constitutive expression of *PR* genes in transgenic poplar [34]. Here, the promoter of *PtrWRKY89* was isolated and fused with *GUS* reporter gene, then transformed into *Arabidopsis*. When treated with low concentration of SA and SA+MeJA mixture, transgenic *Arabidopsis* showed a significant induction of GUS activity but no GUS activity was detected upon treatment with MeJA alone, indicating *PtrWRKY89* was involved in synergistic interactions between the SA and JA signaling pathways. The overexpression lines of *PtrWRKY89* showed more susceptibility to both hemibiotrophic and necrotrophic pathogens, such as *P*. *syringae* and *B*. *cinerea*. Quantitative real-time PCR (qRT-PCR) analysis showed reduced expression of SA and JA pathway-related genes in transgenic plants. These results indicated that PtrWRKY89 could negatively regulate both SA- and JA-related signaling pathways.

## Methods and Materials

### Plant Material and Treatments

Seedlings of *Arabidopsis thaliana* (Columbia 0 ecotype) were transferred to pots after 2 weeks of germination on plates of MS medium [35]. Plants were grown on a 1:1 mixture of peat and vermiculite in an illumination incubator at 22°C, 80% relative humidity and a 16 h photoperiod with supplemental light [36]. *Populus trichocarpa* Torr. and A. Gray was grown in a greenhouse at 25°C under a 14 h light with supplemental light (4,500 lux) and 10 h dark cycle. 2-month-old plants were prepared for gene expression analyses [36].

SA (100 μM), MeJA (100 μM) and SA+MeJA mixture were used to whole plant of *Arabidopsis* as described by Mur et al (2006). Inoculations *Pseudomonas syringae* pv. tomato DC3000 strain (OD600 = 0.001 in 10 mM MgCl_2_) were performed by infiltration of leaves and the inoculated leaves were harvested at the 3^rd^ day after infiltration and homogenized in 10 mM MgCl_2_. Diluted extracts of leaf were plated on King’s B medium with rifampicin (100 mg/mL), incubated at 25°C for 2 days and counting the colony forming units. Every sample of diluted extracts was repeated on three plates. *Botrytis cinerea* was cultured, collected and suspended the spores, then sprayed the spore suspending on to plants, which was performed as described previously [31].

### Cloning of the Promoter Fragment of *PtrWRKY89* and Transformation of *Arabidopsis*

A 1422-bp upstream sequence of *PtrWRKY89* was amplified using *P*. *trichocarpa* genomic DNA as template by the primers: ProPtrWRKY89-F: 5'- TAAAGTGATACAGGGATGGT-3'; ProPtrWRKY89-R: 5'- TACCTTCTTTCTATGTGAGG-3'. The PCR reaction was performed using PrimerSTAR HS DNA Polymerase (TaKaRa, Dalian, China) in a total volume of 50 μL. The PCR conditions used were as follows: 98°C for 3 min; 32 cycles of 98°C for 30 s, 60°C for 30 min and 72°C for 2 min, followed by a final extension of 72°C for 10 min. The promoter fragment of *PtrWRKY89* was inserted into the binary vector pCXGUS-P [37] to drive *GUS* expression. The resulting construct was transferred into GV3101, an *Agrobacterium tumefaciens* strains by the freeze-thaw method.

Transformation of *A*. *thaliana* plants was performed according to the floral dip method [38]. Transformants were screened on MS plates supplemented with 30 mg/mL of hygromycin and 50 mg/mL of cefotaxime sodium to inhibit growth of agrobacterium. Finally, genomic DNA was extracted from leaves of transgenic plants by a cetyl trimethyl ammonium bromide (CTAB) method [39]. A 20 ng sample of genomic DNA from each plant were applied as the template to determine the integration of the transgene by PCR. Each PCR mixture contained 5 μL GoTaqRGreen Master Mix (Promega, Beijing, China), 0.5 μL cDNA, 0.25 μL of each primer and 4 μL nuclease-free water.

### RNA Extraction and Semi-Quantatitive RT-PCR and qRT-PCR

Total RNA from plant tissues was extracted by RNA RNeasy Plant Mini Kit (Qiagen, Germany). Samples from at least three plants were mixed for RT-PCR analysis. Total RNA was treated with RNase-free DNase (TaKaRA, Dalian, China) to avoid genomic DNA contamination. First-strand cDNA was generated from 2 μL RNA with RT-AMV transcriptase (TaKaRa, Dalian, China) in the volume of 20 μL with oligo (dT)18 at 42°C for 30 min. The products of RT-PCR were resolved by 1% (W/V) agarose gel electrophoresis and stained in EB solution, then visualized with under UV light to determine the expression level of *PtrWRKY89* in transgenic plants and *18S rRNA* was used as an internal control. qRT-PCR analysis was performed in a total volume of 25 μL containing 12.5 μL of SYBR Premix ExTaqTM (TaKaRa, Dalian, China), 0.5μL of each primer, 1μL cDNA and 10.5μL nuclease-free water. Differences of genes expression were calculated using the [Δ][Δ]C_t_ = 2^[Δ]Ct,18S-[Δ]Ct^ method compared to the control and *Arabidopsis UBC* gene was used as an internal control. The gene-specific primers used for semi-qRT-PCR and qRT-PCR were shown in S1 Table. The analysis of qRT-PCR was built on at least two biological replicates of each sample with three technical replicates.

### Subcellular Localization and Transactivation Assay

*PtrWRKY89* fragments were amplified by PCR applying the primers (PtrWRKY89-SL-F and PtrWRKY89-SL-R) with PrimerSTAR HS DNA Polymerase (TaKaRa, Dalian, China) and ligated into pCX-DG [37] to generate the *35S-GFP*:*PtrWRKY89* construct and the resulting vector was shot into cells of onion epiderm by Gene Gun (GJ-1000, SCIENTZ, China). The cell nucleus of onion epidermal cells was highlighted by DAPI staining and visualized under a light microscope (Olympus BX53).

The amplification of *PtrWRKY89* ORF was performed by PCR with primers PtrWRKY89-yeast-F: 5'CGCGGATCCAGATGGAGTCTTCTTGGCCTGAG3' and PtrWRKY89-yeast-R: 5'AAAACTGCAGGGAAAATACAAAGAGGCTGC3' and cloned into *Bam*HI*/Pst*I-digested pGBKT7 vector. The resulting vector and empty vector pGBKT7 (negative control) were transformed into yeast strain *Saccharomyces cerevisiae* Gold2, respectively by PEG-LiAc method. Transformants were screened on synthetic dropout medium (SD medium) lacking tryptophan (Trp) and then transported on SD medium without Trp, histidine (His) and adenine (Ade) for the transactivation analysis according to the method described previously [40].

### GUS Staining

The method of GUS staining was described as Duan et al (2015) and the staining solution contained 0.1 M sodium phosphate buffer, pH 7.0, 2 mM K_3_Fe(CN)_6,_ 2 mM K_4_Fe(CN)_6_, 0.2% Triton X-100, 2 mM X-Gluc, 10 mM EDTA [36].

### DAB Staining

DAB staining were performed as previously described [41].

### Extraction and Measurement of Chlorophyll

200 mg of leaf tissue was homogenized with an acetone:dimethyl sulphoxide (DMSO) (1: 1 V/V) mix for extraction of total chlorophyll as described previously [42]. The absorbance of supernatant was recorded at 663 and 645 nm using UV-visible spectrophotometer Model DU800 (Shimadzu Corporation, Kyoto, Japan). Based on the method described previously, total chlorophyll was measured [43]. At least three plants for each independent line were examined and all tests were conducted by performing three technical replicates for each sample.

## Results

### Expression Profiles of the *PtrWRKY89* Promoter in Transgenic *Arabidopsis*

**Fig 1:**
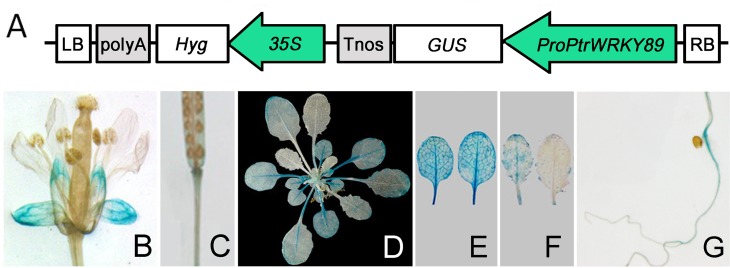
Expression analysis of the *PtrWRKY89* promoter. (A) The promoter of *PtrWRKY89* was cloned and ligated into vector pCXGUS-P to drive *GUS* expression and the resulting construct was introduced into *A*. *thaliana*. Transgenic seedlings were grown on MS media and then transplanted in soil for GUS staining. *GUS* expression was observed in various tissues of transgenic plants, including (B) flowers, (C) siliques, (D) 5-week-old seedlings, (E) old leaves, (F) young leaves and (G) roots.

Our previous study has shown that *PtrWRKY89* rapidly induced by SA and enhanced resistance accomplished with increased pathogenesis-related protein genes (*PR*s) in *Populus* [34]. In order to investigate the spatial and temporal expression patterns of *PtrWRKY89*, a 1,422-bp promoter fragment of *PtrWRKY89* (*ProPtrWRKY89*) was isolated from the genome of *P*. *trichocarpa* and fused with *GUS* reporter gene to generate the *ProPtrWRKY89-GUS* construct (Fig 1A), which was then introduced into wild-type *Arabidopsis thaliana* (WT). Histochemical GUS staining of transgenic *Arabidopsis* showed that GUS activity was detected in calyx and slightly at the conjunction area of siliques, carpopodium and root (Fig 1B, 1C and 1G). In addition, high GUS expression levels were observed in old leaves but not in young leaves (Fig 1D–1F). These results were consistent with the expression patterns of *PtrWRKY89* in poplar leaves at different developing stages (S1 Fig).

### *PtrWRKY89* Was Induced by Low Concentration SA and JA

**Fig 2:**
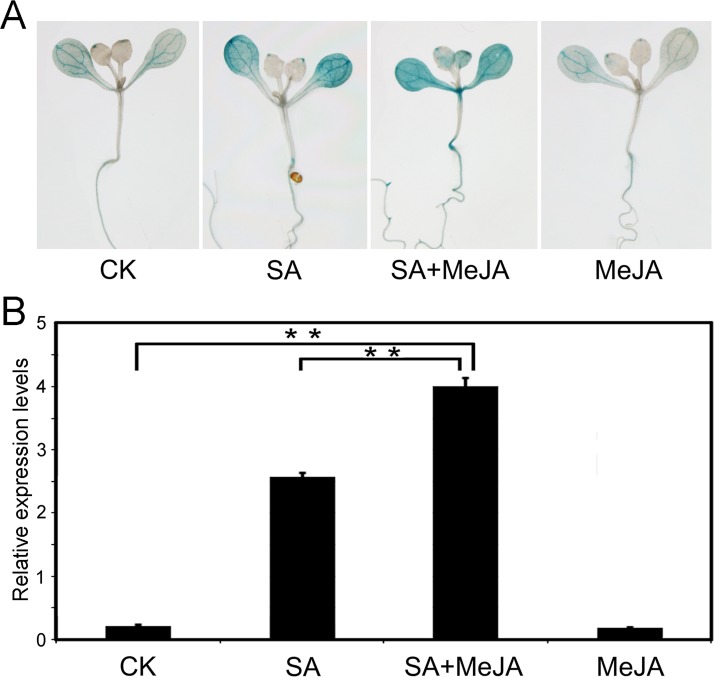
Expression patterns of the *PtrWRKY89* promoter driven *GUS* gene in response to salicylic acid (SA), methyl jasmonate (MeJA) with low concentration and their mixed solution, respectively. SA solution (100 μΜ), MeJA solution (100 μΜ) and SA+MeJA mixture (100 μΜ + 100 μΜ) were sprayed onto the surface of two-week-old transgenic *Arabidopsis* seedling containing the *ProPtrWRKY89-GUS* construct. The control plants (CK) were treated by H_2_O. After treatments of 24 h, the seedlings were collected from MS medium. (A) The GUS staining of the seedlings. (B) The transcript profiles of *PtrWRKY89* were analyzed by quantitative RT-PCR (qRT-PCR). Error bars were obtained from three biological replicates. *Arabidopsis UBC* (AT5G25760) expression was used as a control and gene-specific primers were used for qRT-PCR analysis were described in S1 Table. Two asterisks indicate a statistically significant difference by Student's *t*-test (**, *P* < 0.01).

Previously, *PtrWRKY89* was demonstrated to be induced rapidly by 5 mM SA solution and slightly induced by 1 mM MeJA solution [34]. In order to test the expression level of *PtrWRKY89* under lower concentration SA or MeJA, the *Arabidopsis* plants containing the *ProPtrWRKY89-GUS* construct were sprayed by 100 μM SA and MeJA, respectively. As shown in Fig 2, SA could impressively induce *GUS* expression but MeJA did not cause any significant changes compared to the control line. It has been reported that application of low concentrations of SA and JA mixture could exert the antagonistic effect between SA and JA signals to induced *PR*s and *PDF1*.*2* simultaneously [18]. We wondered whether PtrWRKY89 potentially has a role in controlling both SA- and JA-dependent defense signaling under low concentration of SA and JA. As shown in Fig 2, *GUS* expression was significantly induced by 100 μM SA and higher expression was detected when treated with mixed solution with 100 μM SA and 100 μM MeJA mixed solution (SA+MeJA), compared to the control Interestingly, the mRNA level of *GUS* expression did not significantly altered upon treatment with MeJA alone. These results indicated that PtrWRKY89 might be involved in regulated the interaction between the SA and JA signaling pathways.

### PtrWRKY89 Acts as a Potential Transcriptional Activator in the Nucleus

**Fig 3:**
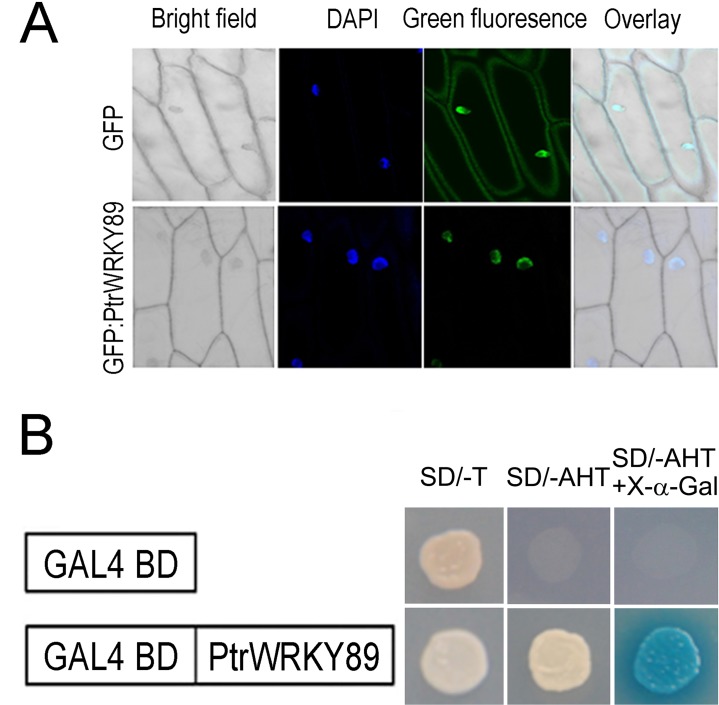
Subcellular localization and transactivation assay of PtrWRKY89. (A) *PtrWRKY89* was ligated into pCX-DG vector to generate *GFP*:*PtrWRKY89* construct. The resulting construct and empty vectors were transformed into epidermal cells of onion (*Allium cepa*) and stained with DAPI, respectively. The fusion protein displayed its localization to the cell nucleus as manifested by GFP (lower column) and *GFP* driven by the *CaMV 35* promoter was localized to both the cytoplasm and the cell nucleus (upper column). Overlay and bright field images of the epidermal cells were also shown. (B) *PtrWRKY89* was cloned into pGBKT7 vector with DNA binding domain of GAL4 and introduced into Gold2 yeast cells. The transformants were grown on SD medium lacking tryptophan (Try) for the sake of positive clone selection and then on SD medium without Try, histidine (His) and adenine (Ade) for the transactivation assay. The clones grown on SD (-Try/His/Ade) were stained by X-α-gal. GAL4-BD (empty vector) was a negative control.

A putative nuclear localization signal (NLS) was predicted at the N-terminal of the PtrWRKY89 protein [34]. In order to verify the subcellular localization, the *PtrWRKY89* and green fluorescent protein (GFP) genes were fused together and controlled by the constitutive *CaMV 35S* promoter to generate *35S-GFP*:*PtrWRKY89* construct, and the construct was transformed into onion epidermal cells by bombardment. The result showed that the GFP signal was observed only in the nucleus of cells transformed *35S-GFP*:*PtrWRKY89* construct (Fig 3A), while in *35S-GFP* control lines, GFP fluorescence was present throughout the cytoplasm and the nucleus (Fig 3A). These results indicated that PtrWRKY89 is localized in the nucleus.

To determine whether PtrWRKY89 functioned as a transcription factor, *PtrWRKY89* ORF was cloned into pGBKT7 vector to fuse with the GAL4 DNA-binding domain, following transformed into Gold2 yeast (*Saccharomyces cerevisiae*) cells. As shown in Fig 3B, the transformants containing pGBKT7-PtrWRKY89 could grow well on selective medium lack of tryptophan (SD/-Trp) and the medium without tryptophan, adenine and histidine (SD/-Trp/-Ade/-His). In contrast, the yeast cells transformed pGBKT7 grew on SD/-Trp medium, but not on SD/- Trp/-Ade/-His (Fig 3B). These results demonstrated that PtrWRKY89 acts as a transcriptional activator.

### Overexpression of *PtrWRKY89* in *Arabidopsis* Resulted in Reduced Resistance against *Pst*DC3000

**Fig 4:**
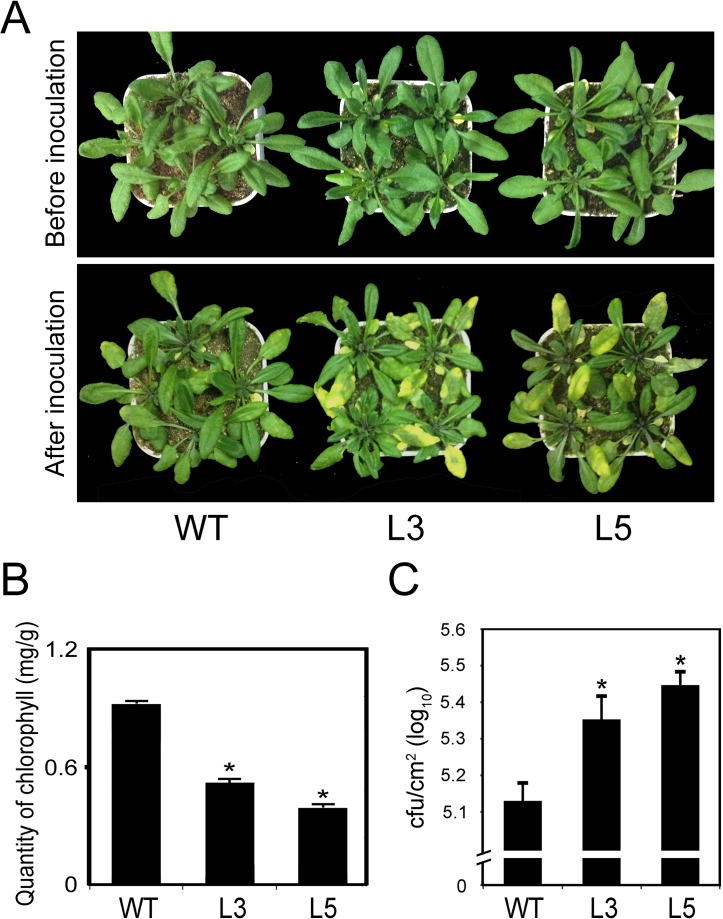
*PtrWRKY89* overexpressing *Arabidopsis* plants showed more susceptible to *Pst*DC3000 than wild-type plants. (A) Disease symptoms of wild-type (WT) and transgenic plants after 3 days of *Pst*DC3000 infection. (B) The quantification of total chlorophyll content in transgenic and WT plants at 3 days post infection. (C) Growth of *Pst*DC3000 in *planta* 3 days after inoculation. Values represent means of three replicates. Error bars indicate standard deviation. Asterisks indicate a statistically significant difference between WT and transgenic plants (*, *P*<0.05 by Student’s *t* test).

To determine the potential role of *PtrWRKY89* in interaction with SA and JA signaling, we construct an overexpression vector, in which the ORF of *PtrWRKY89* was driven by *CaMV 35S* promoter [34] and transformed it into *Arabidopsis*. As shown in S2 Fig, transgenic plants with high transcript level of *PtrWRKY89* showed a similar phenotype to WT plants. The transgenic lines and WT plants were inoculated with *Pseudomonas syringae* pv. tomato DC3000 (*Pst*DC3000). After 3 days, the leaves of transgenic lines turned yellow and withered, but slightly symptom was observed in the WT (Fig 4A). The quantification of chlorophyll also showed that less content was in transgenic lines than WT (Fig 4B). In addition, the growth of *Pst*DC3000 in the overexpression lines was significantly higher than the WT plants (Fig 4C). These results indicated that *PtrWRKY89* overexpression led to reduced resistance against *Pst*DC3000 in transgenic *Arabidopsis*.

### Constitutive Expression of *PtrWRKY89* in *Arabidopsis* Caused More Susceptibility to *Botrytis cinerea*

**Fig 5:**
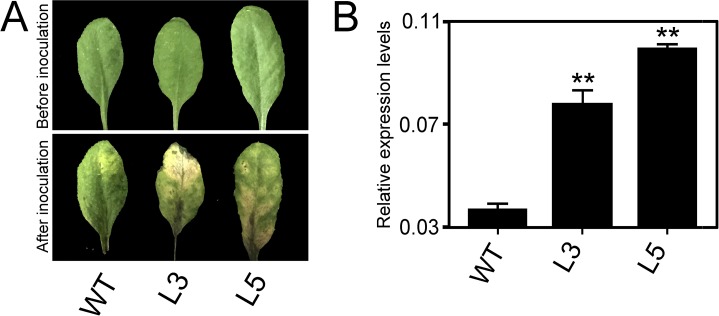
Constitutive expressing *PtrWRKY89* in *Arabidopsis* plants showing susceptibility to *Botrytis cinerea*. (A) Disease response of inoculated plants at 7 days after *B*. *cinerea* infection. (B) Transcript accumulation of *B*. *cinerea Actin* gene in these inoculated plants. *Arabidopsis UBC* gene was used as an internal control. Values represent means of three replicates and error bars indicated standard deviation. Two asterisks indicated a statistically significant difference between WT and transgenic plants (**, *P*<0.01 by Student’s *t* test).

In order to test the resistance of transgenic plants against necrotrophic pathogens, the transgenic *Arabidopsis* plants overexpressed *PtrWRKY89* were sprayed by the spore suspending of *B*. *cinerea*. As shown in Fig 5, transgenic lines tested showed a severe necrosis and the lesion areas were more and larger than WT (Fig 5A). Additionally, the transcript levels of *Botrytis Actin* gene were used to indicate fungal growth in *planta* by semi-qRT-PCR and the result showed that significantly increased levels of fungal DNA were detected from the transgenic lines compared to the WT control (Fig 5B). These results indicated that constitutive expression of *PtrWRKY89* resulted in more susceptibility to *B*. *cinerea*.

### Expression Levels of SA- and JA-Related Genes Were Affected in Transgenic *Arabidopsis* Constitutive Expressed *PtrWRKY89*

**Fig 6:**
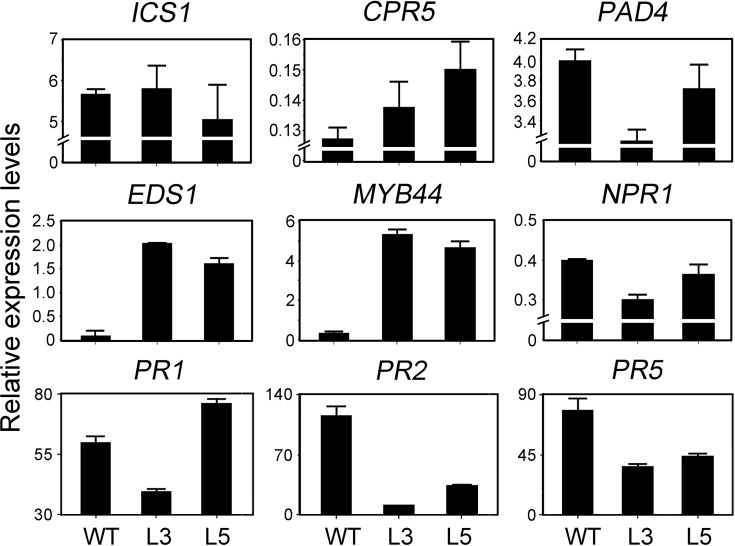
Expression of SA-related genes in transgenic *Arabidopsis* overexpressed *PtrWRKY89* after inoculation of *Pst*DC3000. *ICS1*: *ISOCHORISMATE SYNTHASE 1*, *PAD4*: *PHYTOALEXIN DEFICIENT 4*, *EDS1*: *ENHANCED DISEASE SUSCEPTIBILITY 1*, *MYB44*: *ARABIDOPSIS THALIANA MYB DOMAIN PROTEIN 44*, *NPR1*: *NONEXPRESSER OF PR GENES 1*, *CPR5*: *CONSTITUTIVE EXPRESSION OF PR GENES 5*, *PR1/PR2/PR5*: *PATHOGENESIS RELATED GENES 1/2/5*. *Arabidopsis UBC* gene was used as an internal control.

The expression of genes involved in SA pathway after inoculation of *Pst*DC3000 was tested. As shown in Fig 6, *ISOCHORISMATE SYNTHASE 1* (*ICS1*) which is involved in SA synthesis [1] has no significant changes both in WT and transgenic plants. *PHYTOALEXIN DEFICIENT 4* (*PAD4*), a lipase-like gene that is important for SA signaling and basal plant disease resistance [44], displayed decreased mRNA level in Lines L3 and L5. *ENHANCED DISEASE SUSCEPTIBILITY 1* (*EDS1*) with homology to eukaryotic lipases, functioned upstream of SA-dependent *PR1* mRNA accumulation [45], had an increasing tendency in constitutive expressing *PtrWRKY89* plants. The transcript level of *MYB44* which acts as a transcription activator of SA signaling pathway [22], also enhanced in the transgenic lines compared to WT. *NONEXPRESSER OF PR GENES 1* (*NPR1*) also reduced the mRNA level in the transgenic lines. The expression level of *CONSTITUTIVE EXPRESSION OF PR GENES 5* (*CPR5*), whose mutant showed constitutive expression of systemic acquired resistance (SAR) [46], was increased in the transgenic plants. The marker genes of SA signaling pathway, *PR2* and *PR5* had severe low mRNA levels in transgenic lines than the WT, meanwhile *PR1* had no obviously different. In addition, other SA related genes, such as *PAL1/PAL2/PAL3/PAL4*, *EDR1*, *WRKY60*, *TGA5/6* and *GRX480* were tested by semi-qRT-PCR and showed no significant difference (S3 Fig). These results indicated constitutive expressing *PtrWRKY89* contributed to regulation of SA-related genes and seemed to suppress the activation of SA signaling pathway.

**Fig 7:**
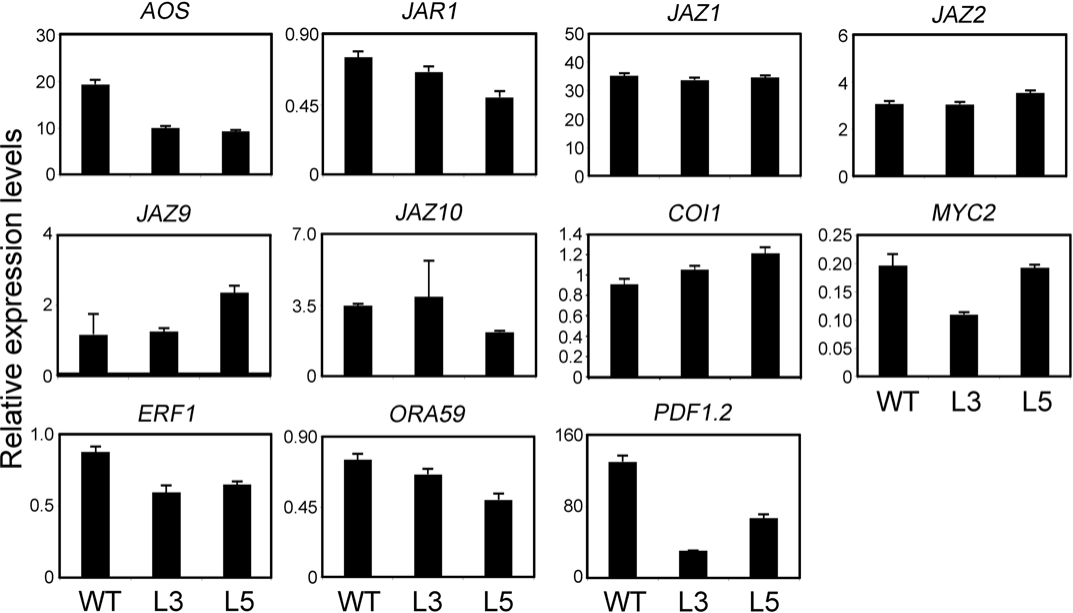
Gene expression analyses of JA-signaling pathway genes in transgenic *Arabidopsis* overexpressed *PtrWRKY89* after spraying spore suspending of *B*. *cinerea*. *AOS*: *ALLENE OXIDE SYNTHASE*, *JAR1*: *JASMONATE RESISTANT 1*, *JAZ1/JAZ2/JAZ9/JAZ10*: *JASMONATE-ZIM-DOMAIN PROTEIN 1/2/9/10*, *COI1*: *CORONATINE INSENSITIVE 1*, *ERF1*: *ETHYLENE RESPONSE FACTOR 1*, *ORA59*: *OCTADECANOID-RESPONSIVE ARABIDOPSIS AP2/ERF 59*, *PDF1*.*2*: *PLANT DEFENSIN 1*.*2*. *Arabidopsis UBC* gene was used as an internal control.

The expression level of the genes related with JA synthesis were also examined, JA signal transduction and JA-induced plant defensin after inoculation of fungi. As shown in Fig 7, *ALLENE OXIDE SYNTHASE* (*AOS*), which was demonstrated to be involved in JA biosynthesis process [47], was reduced transcript levels in the transgenic lines. *JASMONATE RESISTANT 1* (*JAR1*), which catalyzed the formation of a biologically active jasmonyl-isoleucine (JA-Ile) conjugate [8], had lower mRNA level in transgenic *35S-PtrWRKY89* plants. JAZ1, JAZ2, JAZ9 and JAZ10, belonging to jasmonate-zim-domain protein family which acted negatively to repress JA signal [48], had no significant changed transcript levels in the transgenic plants compared to WT. *CORONATINE INSENSITIVE 1* (*COI1*), required for JA signal transduction [49], had a slight increase in transgenic lines. MYC2, a basic helix-loop-helix (bHLH) transcription factor recognized the G-box in the promoter of target genes and orchestrated different branches of the JA pathway [7]. In overexpression line L3, the expression level of *MYC2* was lower than that in WT, but no significant reduction in the L5 (Fig 7). Expression of two ERF/AP2 transcription factors, *ETHYLENE RESPONSE FACTOR 1* (*ERF1*) and *OCTADECANOID-RESPONSIVE ARABIDOPSIS AP2/ERF 59* (*ORA59*) integrating JA and ethylene signals, and regulating some of the MYC2-modulated responses in an opposite fashion [7], was down-regulated compared to WT plants (Fig 7). *PLANT DEFENSIN 1*.*2* (*PDF1*.*2*), encoding a JA-responsive plant defensing was suppressed in the transgenic lines (Fig 7). Additionally, *VSP1/2* and *COR1*, which are induced by JA signaling have no difference between WT and transgenic plants (S3 Fig). These results indicated that overexpression of *PtrWRKY89* also affected JA signal in *Arabidopsis*.

## Discussion

Plants are sessile organisms which demand to accommodate their constantly changes in environmental conditions. The specific plant response to a particular stimulus which is important for its fitness and survival is mediated by a complicated hormonal network [7]. Salicylic acid (SA) and jasmonic acid (JA) signaling pathways, acting opposite functions, play crucial roles in response to diverse lifestyle pathogens in plants [50]. The SA-dependent pathway mainly confers resistance to biotrophic and hemibiotrophic pathogens, such as *Pseudomonas syringae* pv. tomato DC3000 (*Pst*DC3000) [20]. On the other hand, JA-mediated signaling pathway was mainly linked to resistance to necrotrophic fungi like *Botrytis cinerea* [20]. Although plants orchestrate SA and JA pathways to resist pathogens with different lifestyles, *Arabidopsis* and poplar evolved discriminating models to survive from invasion. For example, poplar developed more defense-related genes than *Arabidopsis* to ensure sufficient genes supporting the defense process. Following, the functions of ortholog genes distinguished in these two species, for instance, *Arabidopsis AtWRKY23* was demonstrated to be involved in resistance to nematode infection and responsive to auxin [51] but the *PtWRKY23* from poplar was induced by SA and negative effected resistance to rust infection [52]. Further, the species-specialized genes participate in defense, for example, *Arabidopsis PLANT DEFENSIN 1*.*2* (*PDF1*.*2*) is impressively induced by JA and necrotrophic fungi but we could not find its ortholog in the genome of poplar indicating something different in JA pathway in these two species and poplar adopted specialized JA marker genes to respond JA signaling. By contrast, the SA marker genes *PATHOGENESIS-RELATED* (*PR*s) could be induced by rust infection in poplar, a biotrophic pathogen [52, 53], which is consistent with our previous result [34].

Increasing evidences demonstrated plants recruit transcription factors in modulating these two pathways, such as WRKY transcription proteins. Among WRKY members, AtWRKY70 belonging to Group III was rapidly induced by SA treatment and functioned downstream of *NPR1* [24]. Overexpression of *AtWRKY70* resulted in more resistant to *Pst*DC3000 and susceptible to *Alternaria brassicicola*, accompanied with constitutive expressing *PR1* and suppressed *PDF1*.*2*, respectively [24, 54]. The double mutant of *atwrky70* and *atwrky54*, which was the homologue of AtWRKY70, showed higher level of SA content, indicating that AtWRKY70 and AtWRKY54 were the repressors of SA biosynthesis [55]. Recent research showed that *AtWRKY70* was involved in leaf senescence because the expression level was increasing in the old leaves [56]. A previous study has shown that *PtrWRKY89* was induced by SA treatment in *Populus trichocarpa* and overexpressing *PtrWRKY89* caused increased expression of pathogenesis related genes (*PR*s) [34]. In this study, we found that *PtrWRKY89* gene expression was high in old leaves but not in young leaves in poplar and transgenic *Arabidopsis* containing *ProPtrWRKY89-GUS* construct (Fig 1 and S1 Fig). However, further studies revealed that overexpressing *PtrWRKY89* reduced resistance to SA- and JA-mediated defense pathways to results in more sensitive to *Pst*DC3000 and *B*. *cinerea*, indicating that AtWRKY70 and PtrWRKY89 could play different roles in *Arabidopsis*. In addition, relative low identity of amino acid sequences between AtWRKY70 and PtrWRKY89 also indicated that their biological functions are similar but different [34].

SA and JA signaling pathways usually interact antagonistically to regulate plant defense pathways, but simultaneous exertion of low concentration SA and JA could induce SA- and JA-responsive gene expression, such as *PR1* and *PDF1*.*2*, causing plant cell death [18]. Two *atwrky70* mutants showed enhanced expression level of *PR1* and *PDF1*.*2*, indicating that AtWRKY70 was associated with synergic effect between SA and JA [33]. In order to investigate the potential roles of PtrWRKY89, in synergic interaction between the SA and JA signaling mechanisms, SA and MeJA mixed solution with low concentration were applied to spray transgenic *Arabidopsis* containing the *GUS* gene driven by the promoter of *PtrWRKY89*. The result showed that *GUS* expression was significantly induced by the mixture compared to the control and even higher than it was induced by SA alone (Fig 2). Interestingly, MeJA treatment did not significantly induce *PtrWRKY89* expression level, indicating that PtrWRKY89 might be involved in synergic interactions of SA and JA pathways rather than simple antagonistic effect.

Li et al (2004) reported that overexpression line of *AtWRKY70* showed smaller size than WT, causing by constitutive expressing SA-related genes involved in plant defense. To further illuminate the function of *PtrWRKY89* in interactions between SA and JA signaling pathways, the coding sequence of *PtrWRKY89* driven by *CaMV 35S* promoter was introduced into *Arabidopsis* (S2A Fig). No obvious change of morphology was observed in transgenic plants compared to WT (Fig 4A and S2B Fig) unlike transgenic *Arabidopsis* overexpressed *AtWRKY70* [24]. Furthermore, there was no H_2_O_2_ accumulation and cell death in transgenic lines (S4 Fig). In order to elucidate the function of *PtrWRKY89*, transgenic plants overexpressed *PtrWRKY89* were inoculated with *Pst*DC3000 and *B*. *cinerea*. As shown in Fig 5, transgenic plants exhibited more susceptible to both *Pst*DC3000 and *B*. *cinerea* and H_2_O_2_ level, upon invasion by pathogens, was slightly decreased compared to WT (S4 Fig). Altogether, these results implicated that PtrWRKY89 weakened plant defenses by suppression of SA and JA signals.

We further examined transcript levels of genes associated with SA and JA synthesis, signaling transduction and pathogen resistance after pathogen inoculation. The pathogenesis-related genes (*PR1*, *PR2* and *PR5*) were considered to be the marker of SA signaling pathway. *PR2* and *PR5* were significantly reduced in transgenic plants overexpressed *PtrWRKY89* compared to WT (Fig 6), indicating that the transgenic lines exhibited weakened SA signaling pathway. No significant changes in expression level of *ICS1*, which is involved in SA synthesis, between WT and transgenic plants (Fig 6) suggested that PtrWRKY89 could not inhibit SA biosynthesis. EDS1 and PAD4 interacted to form a protein complex and played a major role in SA-mediated defense [57]. Transgenic plants showed an increased in *EDS1* transcript level but accompanied with reduced *PAD4* levels (Fig 6), implying that *PtrWRKY89* overexpression was interfering with SA signaling. CPR5 played as a negative factor of SA pathway [46], hence its enhanced expression level in transgenic plants could reduce the SA signaling (Fig 6).

On the other hand, among the JA-related genes, the marker gene *PDF1*.*2* of JA defense pathway was impressively down-regulated in transgenic lines L3 and L5 compared to WT (Fig 7), indicating the attenuated JA signal. *ERF1* and *ORA59* as the activator of *PDF1*.*2* [7], also reduced mRNA accumulation in transgenic plants (Fig 7). In addition, PtrWRKY89 was a transcription activator to activate expression of target genes (Fig 3), however, other JA and JA-Ile biosynthesis genes, such as *AOS* and *JAR1*, were down-regulated in transgenic *Arabidopsis* overexpressed *PtrWRKY89* (Fig 7). Overall it is indicated that PtrWRKY89 could play as a repressor of SA and JA signaling and we furtherly proposed that PtrWRKY89 reversed the transcription activator to a repressor *via* interaction with transcription repressing proteins in *Arabidopsis*.

## Supporting Information

S1 Fig Expression patterns of *PtrWRKY89* in poplar leaves at the different developmental stages. The leaves at the different developmental stages ranged from the 1^st^ node (1N) to the 8^th^ node (8N), collected from 2-month-old *P*. *trichocarpa*. *18S rRNA* was used as an internal control. Values represent means of three replicates and error bars indicated standard deviation.
(TIF)

S2 Fig Phenotypes of transgenic *Arabidopsis* overexpressed *PtrWRKY89*. Expression levels of *PtrWRKY89* in transgenic *Arabidopsis* analyzed by semi-quantatitive RT-PCR and *18S rRNA* was used as an internal control (Fig A). Two-week-old *Arabidopsis* seedlings were grown on the MS solid medium (Fig B). Transgenic *Arabidopsis* seedlings overexpressed *PtrWRKY89* showed no obvious difference in phenotypes compared to the wild type. No significant difference in phenotypes was observed.
(TIF)

S3 Fig The expression levels of SA and JA related genes in wild type and overexpression *Arabidopsis*. *PAL1/PAL2/PAL3/PAL4*: *PHE AMMONIA LYASE 1/2/3/4*, *EDR1*: *ENHANCED DISEASE RESISTANCE 1*, *WRKY60*: *WRKY DNA-BINDING PROTEIN 60*, *TGA5/6*: T*GACG MOTIF-BINDING FACTOR 5/6*, *VSP1/2*: *VEGETATIVE STORAGE PROTEIN 1/2*, *COR1*: *CORONATINE-INDUCED PROTEIN 1*, these genes were analyzed by semi-quantitative RT-PCR and the primers were listed in S1 Table. The *18S* gene was used as an internal control.
(TIF)

S4 Fig H_2_O_2_ accumulation in transgenic plants in response to *Pst*DC3000 and *B*. *cinerea* inoculation, respectively. The control and inoculated leaves from wild-type and transgenic lines were stained by DAB at 3 days after inoculation of *Pst*DC3000 and 7 days after spraying spore suspending of *B*. *cinerea*, respectively.
(TIF)

S1 Table Primers for semi-qRT-PCR and qRT-PCR in this study. (XLSX)
