# Supplementary figures and images for: Overexpression of Poplar PtrWRKY89 in Transgenic Arabidopsis Leads to a Reduction of Disease Resistance by Regulating Defense-Related Genes in Salicylate- and Jasmonate-Dependent Signaling
Source: PLoS One. 2016 Mar 28;11(3):e0149137. doi: 10.1371/journal.pone.0149137 (PMC4809744; doi:10.1371/journal.pone.0149137)

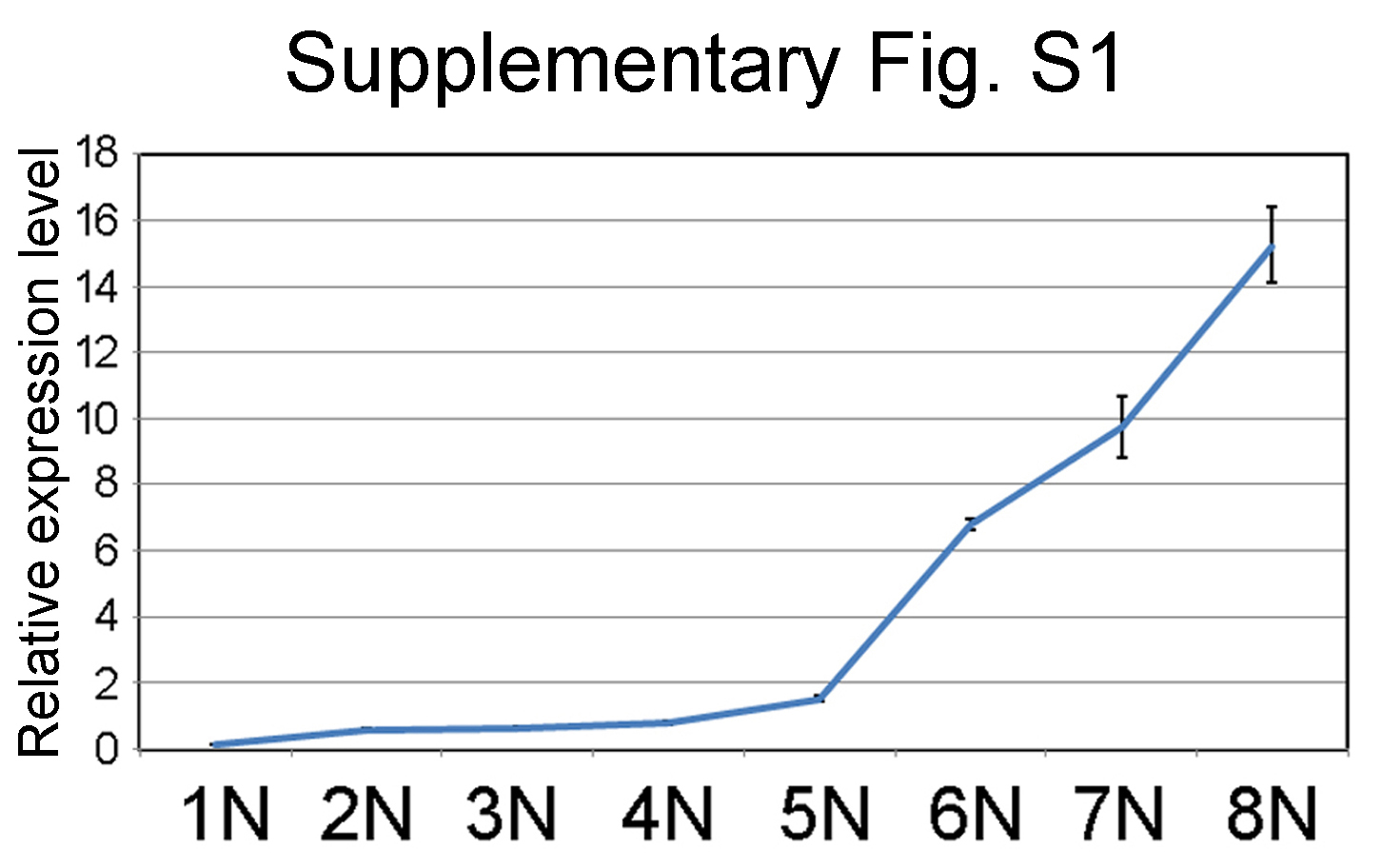

Supplement: S1 Fig — The leaves at the different developmental stages ranged from the 1st node (1N) to the 8th node (8N), collected from 2-month-old P. trichocarpa. 18S rRNA was used as an internal control. Values represent means of three replicates and error bars indicated standard deviation. (TIF) [file pone.0149137.s001.tif]

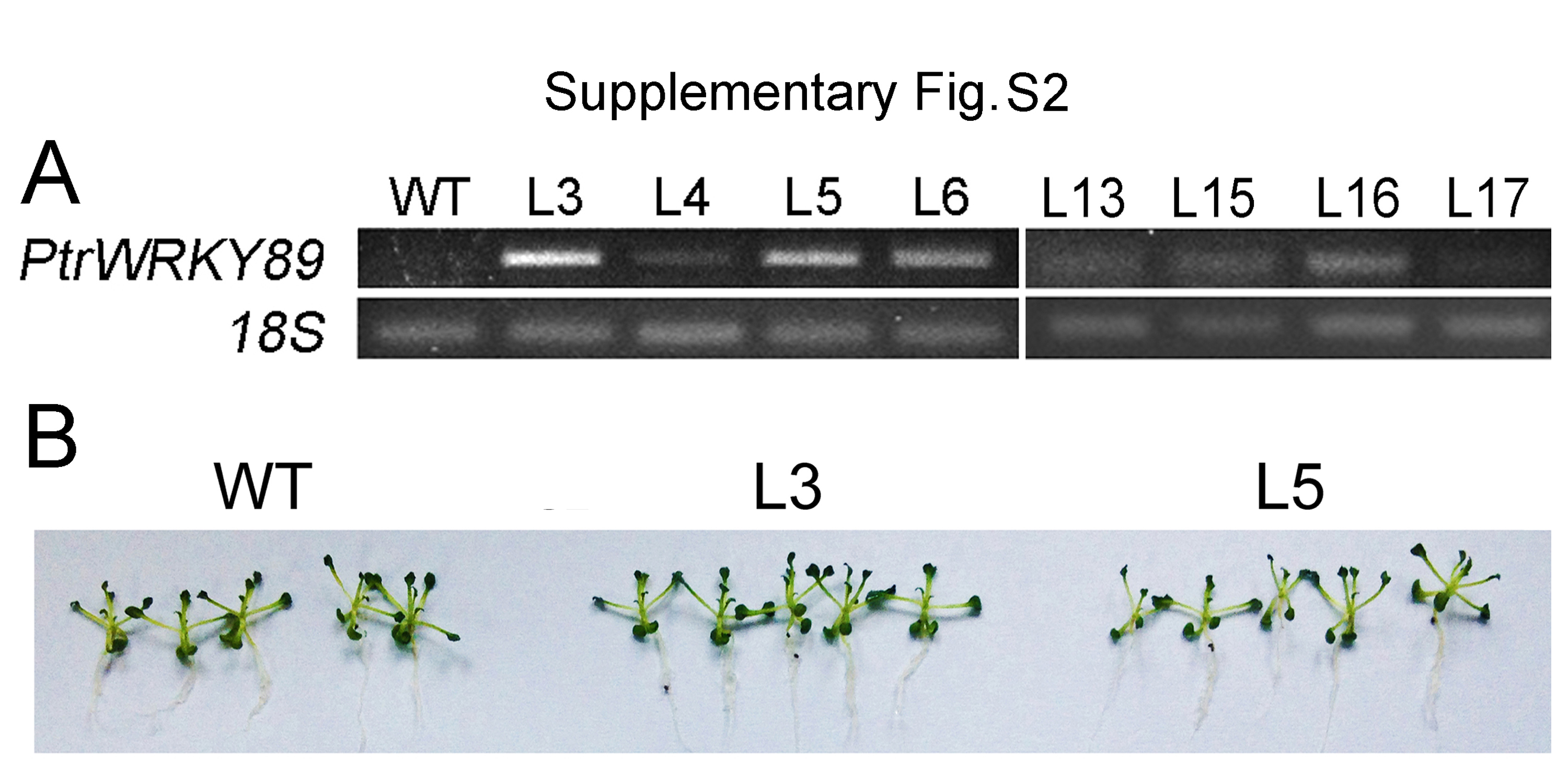

Supplement: S2 Fig — Expression levels of PtrWRKY89 in transgenic Arabidopsis analyzed by semi-quantatitive RT-PCR and 18S rRNA was used as an internal control (Fig A). Two-week-old Arabidopsis seedlings were grown on the MS solid medium (Fig B). Transgenic Arabidopsis seedlings overexpressed PtrWRKY89 showed no obvious difference in phenotypes compared to the wild type. No significant difference in phenotypes was observed. (TIF) [file pone.0149137.s002.tif]

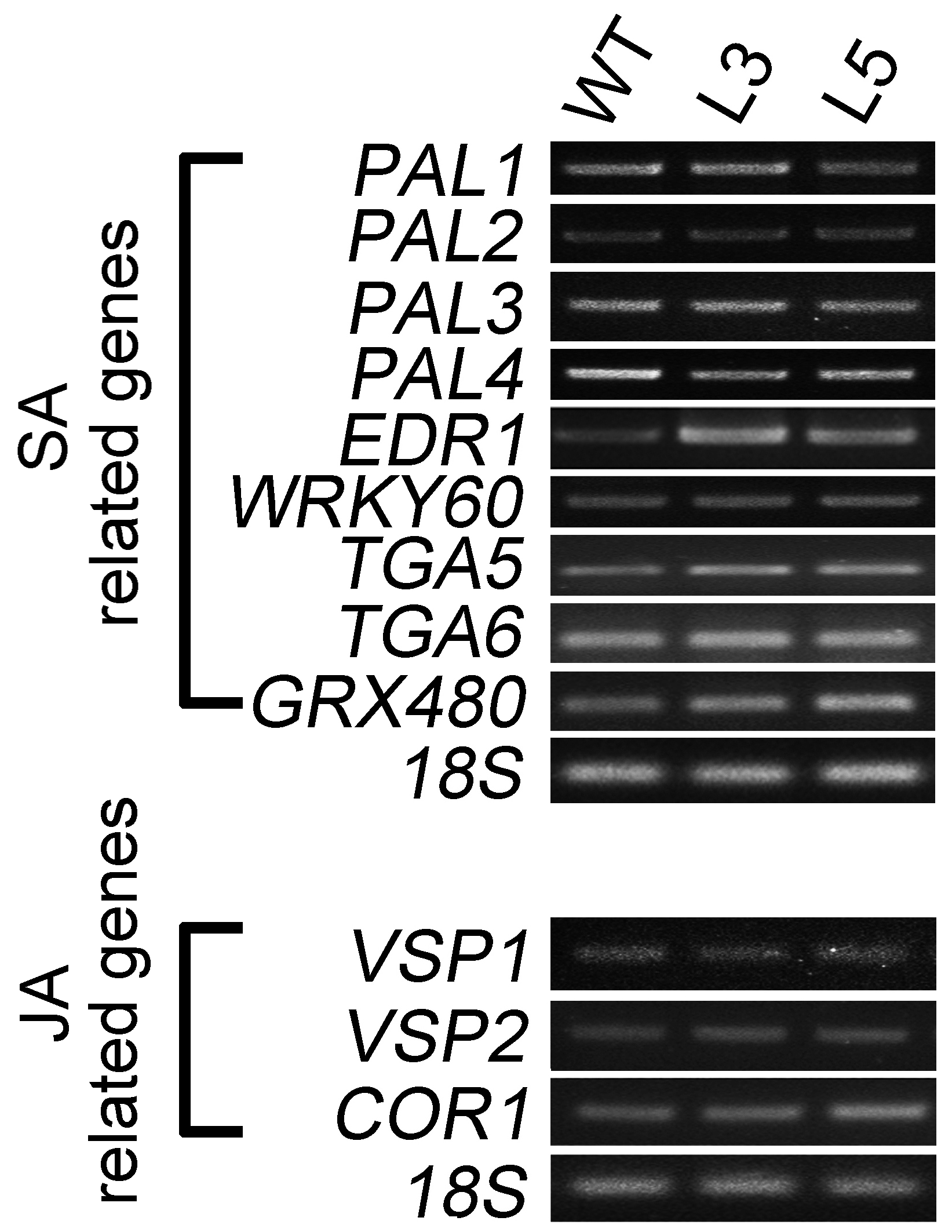

Supplement: S3 Fig — PAL1/PAL2/PAL3/PAL4: PHE AMMONIA LYASE 1/2/3/4, EDR1: ENHANCED DISEASE RESISTANCE 1, WRKY60: WRKY DNA-BINDING PROTEIN 60, TGA5/6: TGACG MOTIF-BINDING FACTOR 5/6, VSP1/2: VEGETATIVE STORAGE PROTEIN 1/2, COR1: CORONATINE-INDUCED PROTEIN 1, these genes were analyzed by semi-quantitative RT-PCR and the primers were listed in S1 Table. The 18S gene was used as an internal control. (TIF) [file pone.0149137.s003.tif]

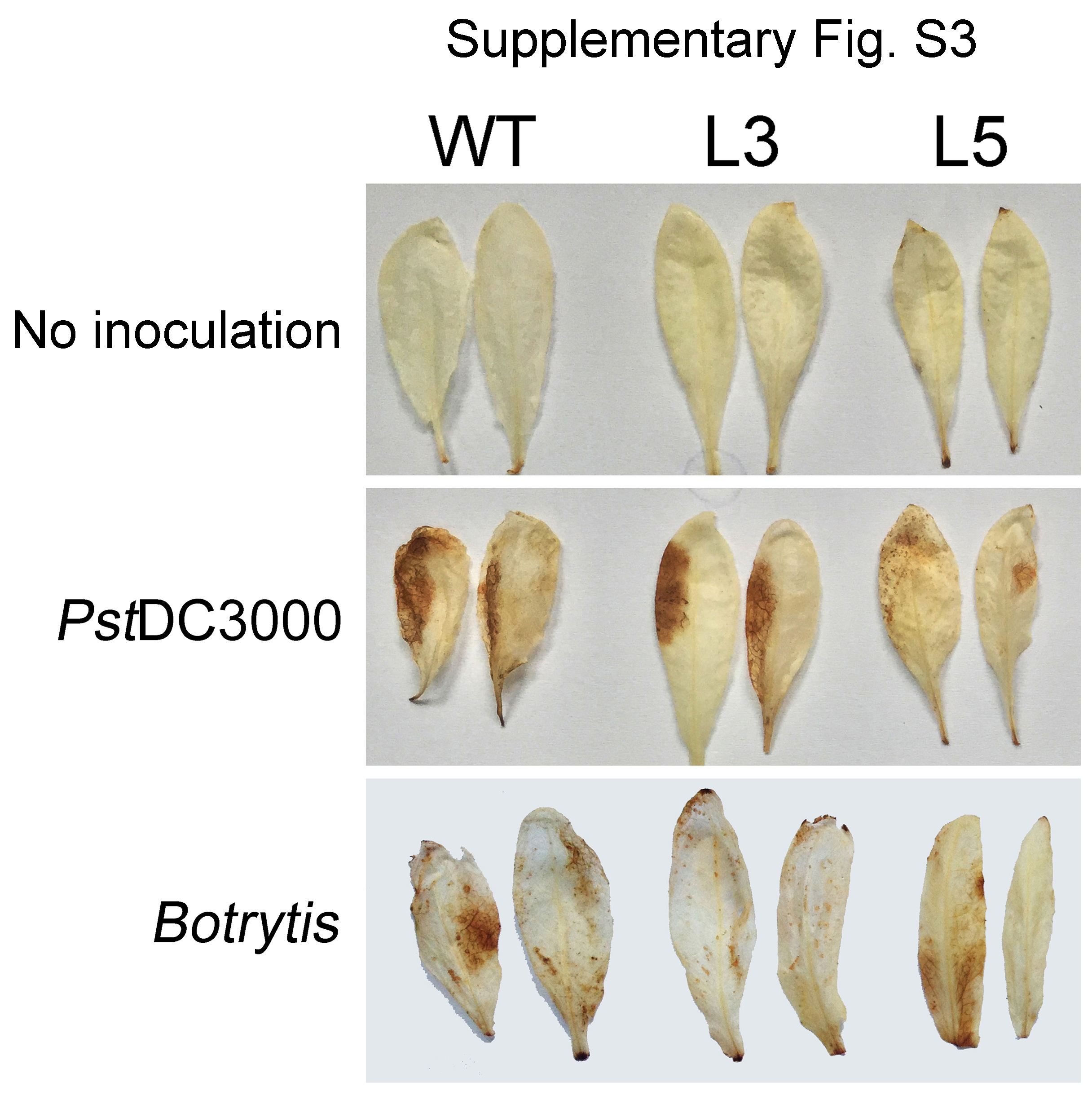

Supplement: S4 Fig — The control and inoculated leaves from wild-type and transgenic lines were stained by DAB at 3 days after inoculation of PstDC3000 and 7 days after spraying spore suspending of B. cinerea, respectively. (TIF) [file pone.0149137.s004.tif]
